# Phosphorylation of the alpha-I motif in SYMRK drives root nodule organogenesis

**DOI:** 10.1073/pnas.2311522121

**Published:** 2024-02-16

**Authors:** Nikolaj B. Abel, Malita M. M. Nørgaard, Simon B. Hansen, Kira Gysel, Ignacio Arribas Díez, Ole N. Jensen, Jens Stougaard, Kasper R. Andersen

**Affiliations:** ^a^Department of Molecular Biology and Genetics, Aarhus University, Aarhus C 8000, Denmark; ^b^Department of Biochemistry and Molecular Biology, University of Southern Denmark, Odense M 5230, Denmark

**Keywords:** root nodule symbiosis, SYMRK, phosphorylation, plant–microbe interaction, plant signaling

## Abstract

Symbiosis receptor-like kinase SYMRK is required for root nodule symbiosis between legume plants and nitrogen-fixing bacteria. To understand symbiotic signaling from SYMRK, we determined the crystal structure to 1.95 Å and mapped the phosphorylation sites onto the intracellular domain. We identified four serine residues in a conserved “alpha-I” motif, located on the border between the kinase core domain and the flexible C-terminal tail, that, when phosphorylated, drives organogenesis. Substituting the four serines with alanines abolished symbiotic signaling, while substituting them with phosphorylation-mimicking aspartates induced the formation of spontaneous nodules in the absence of bacteria. These findings show that the signaling pathway controlling root nodule organogenesis is mediated by SYMRK phosphorylation, which may help when engineering this trait into non-legume plants.

## Significance

Symbiosis between legumes and nitrogen-fixing bacteria is dependent on the Symbiosis receptor-like kinase (SYMRK). Phosphorylation is an important signal for pathway activation, and in this study, we mapped essential phosphorylation sites in the intracellular part of SYMRK. We identify a conserved “alpha-I” motif in SYMRK and demonstrate that phosphorylation of four serine residues in the alpha-I motif drives the cellular program leading to root nodule formation.

Legumes can overcome nitrogen limitations in the soil by acquiring atmospheric nitrogen through symbiosis with rhizobia bacteria (1). Nitrogen-fixing symbiosis requires a highly specific communication pathway where secreted bacterial signals are perceived by plant cell-surface receptors. Nod factors produced by rhizobia are recognized by the LysM receptors NFR1 and NFR5 (2–5) in *Lotus japonicus* (*Lotus*), and these receptors subsequently transduce the signal to the malectin-like/leucine-rich repeat receptor kinase SYMRK (6). Successful establishment of root nodule symbiosis depends on SYMRK, and even spontaneous nodule formation through forced interaction between NFR1 and NFR5 is fully dependent on the presence of SYMRK (7). SYMRK is the first component of the common symbiotic pathway driving symbiosis with rhizobia, *Frankia*, and arbuscular mycorrhizal fungi (8). Here, SYMRK propagates the symbiotic signal to the nucleus of plant root cells, where calcium oscillations are decoded by calcium- and calmodulin-dependent protein kinase (CCaMK) (9, 10), which in turn phosphorylates CYCLOPS to activate the master transcription factor Nodule Inception (NIN) (11). The SYMRK receptor has an active intracellular kinase domain (12), and mutations in the core kinase domain have been linked to loss of symbiotic function (6, 13), showing that kinase activity is important for root nodule formation. Phosphorylation sites in the core kinase domain of *Lotus* SYMRK have been investigated previously; here, S754 and T760 were identified as phosphorylated residues that regulate kinase activity (12). However, it is unknown how phosphorylation outside the kinase core of SYMRK affects symbiotic function. In this study, we determined the structure of the SYMRK kinase domain and identified four phosphorylation sites in a conserved alpha-I motif that drive root nodule organogenesis.

## Results

### Mapping the Phosphorylation Sites onto the SYMRK Kinase Structure.

**Fig. 1.**
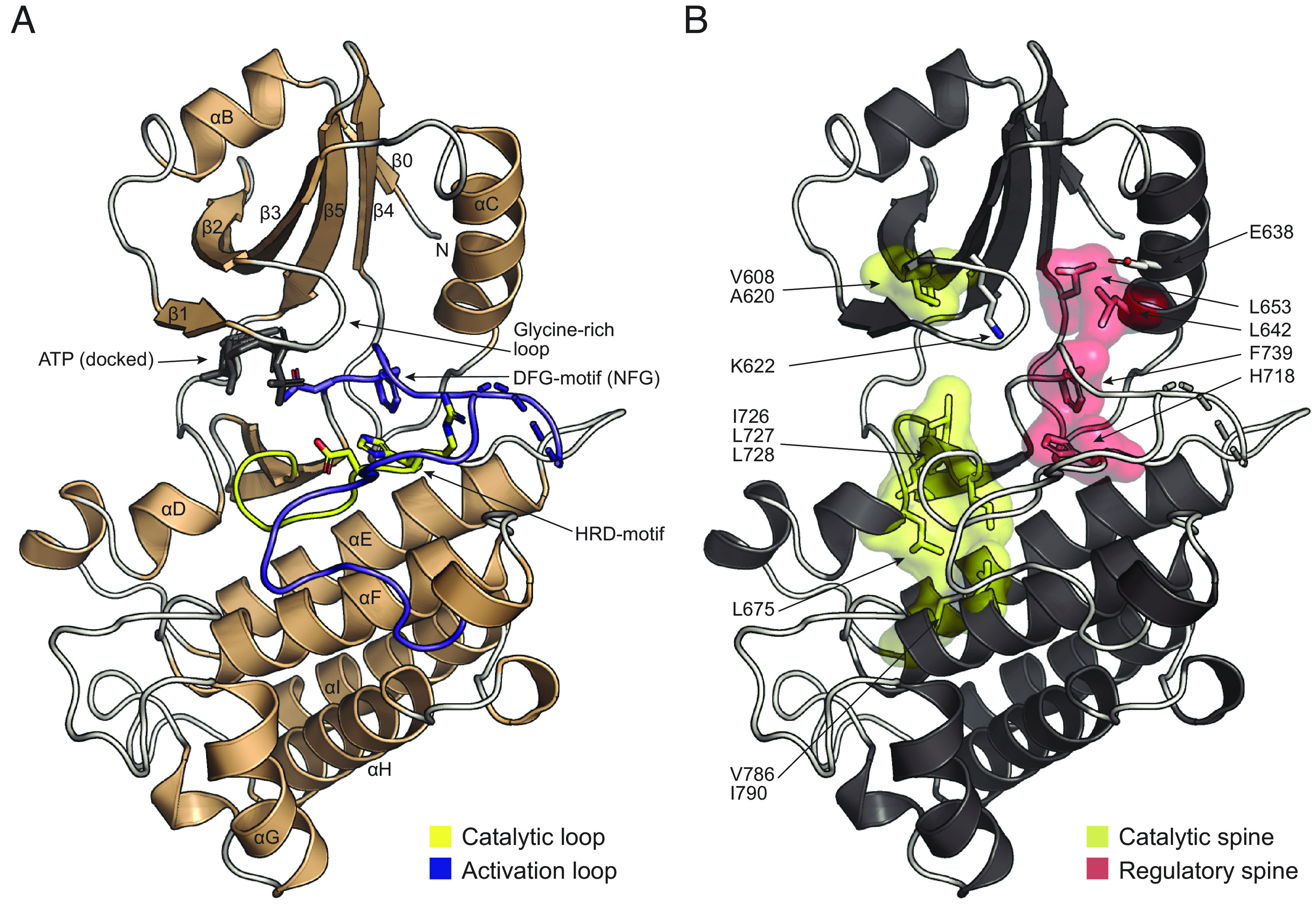
Crystal structure of SYMRK core kinase. (*A*) Cartoon representation of the SYMRK core kinase structure. Conserved secondary structure elements and catalytic/regulatory motifs are indicated. SYMRK was crystallized without adenosine triphosphate (ATP) and to visualize the binding site, an ATP molecule from Protein kinase A (PDB: 1ATP) was docked into SYMRK without sterical clashes. (*B*) The structure of SYMRK is in an inactive state. Important catalytic motifs are highlighted. The C-spine (yellow) is broken due to the lack of bound ATP. The R-spine (red) is assembled, but αC is in an out-position, leaving the salt bridge between the catalytic lysine K622 and αC-glutamate E638 broken.

**Fig. 2.**
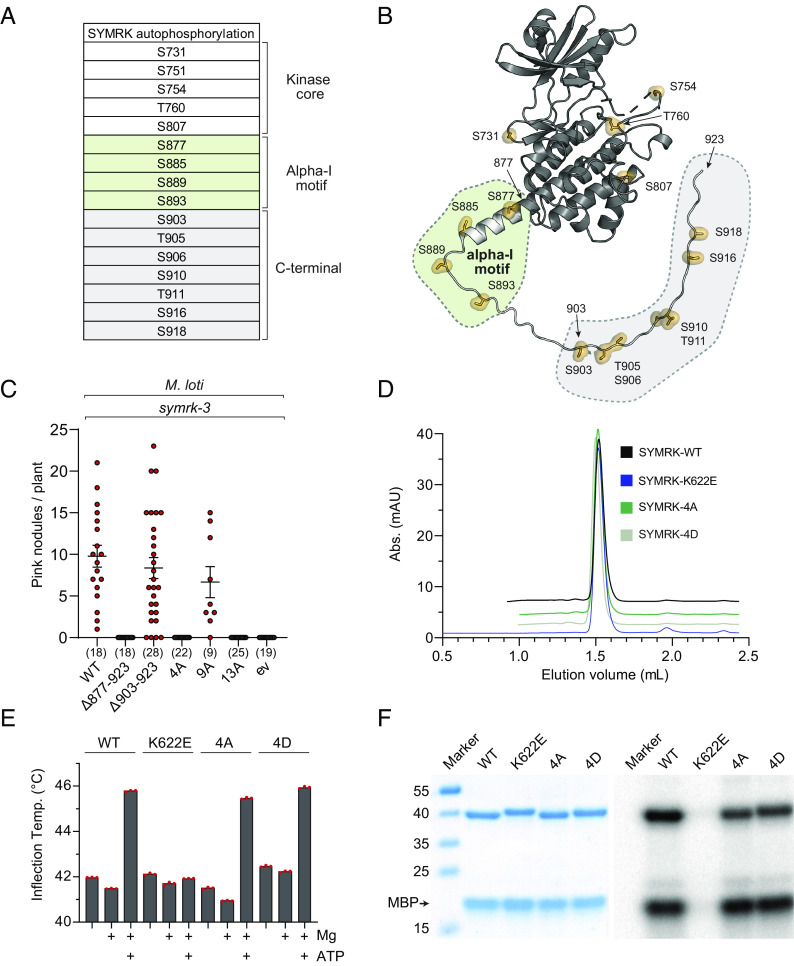
Phosphorylation of the alpha-I motif is essential for root nodule symbiosis. (*A*) List of autophosphorylation sites identified on SYMRK. (*B*) Phosphorylation sites mapped onto the structure of SYMRK. Dark gray is the crystal structure and light gray is the modeled structure (*C*) Number of red infected nodules on hairy roots expressing the indicated SYMRK constructs under the native promotor in *symrk-3* mutant plants, 4 wk after inoculation with *M. loti* rhizobia. ev is a vector without *SymRK*. Numbers below graph specify number of plants for the specific construct. (*D*) Analytic gel filtration profile of SYMRK-WT and SYMRK(-4A, -4D, or -K622E) variants. (*E*) NanoDSF thermal stability assay of SYMRK variants supplemented with 5 mM MgCl_2_ and 1 mM ATP. (*F*) Kinase assay of SYMRK variants incubated with radioactive ATP and MBP substrate to assess both autophosphorylation and transphosphorylation ability. SYMRK and MBP were separated by Sodium Dodecyl Sulfate PolyAcrylamide Gel Electrophoresis (SDS-PAGE) and visualized by Coomassie blue staining (*Left*) and autoradiography (*Right*).

To understand the signaling function of SYMRK in root nodule symbiosis, we expressed, purified, and determined the crystal structure of the core kinase domain (Fig. 1 and *SI Appendix*, Fig. S1*A* and Table S1). A diffraction dataset extending to 1.95 Å was collected, and the phase problem was solved by molecular replacement using a homology model created from the *Arabidopsis thaliana* BIK1 kinase structure (14). The overall crystal structure of the SYMRK kinase domain resembles other eukaryotic kinases of the IRAK4/Pelle-type (15) and *A. thaliana* BAK1 (16) and BIK1. As predicted from the sequence, SYMRK has all the canonical structural motifs required for ATP binding and catalytic activity (Fig. 1*A*). The SYMRK kinase crystallized in an inactive conformation, with a broken catalytic spine (C-spine) due to the lack of a bound nucleotide. However, ATP can easily be docked into its predicted binding site. The regulatory spine (R-spine) is assembled, but the C-helix is in an inactive “out” position, leaving the salt bridge between the catalytic lysine (K622) and αC-glutamate (E638) broken (Fig. 1*B* and *SI Appendix*, Fig. S2). Having established the structure of the archetypal kinase domain, we next investigated possible regulation by phosphorylation to gain insights into how SYMRK signals during root nodule symbiosis. We performed an in vitro autophosphorylation experiment using the complete intracellular domain of SYMRK (*SI Appendix*, Fig. S1*B*). We identified sixteen phosphorylated residues using phosphopeptide enrichment by Zr-IMAC and phosphopeptide sequencing by tandem mass spectrometry (Fig. 2*A* and *SI Appendix*, Table S2) (17). We mapped the phosphorylation sites onto the structure of SYMRK and used Alphafold2 to model the remaining C-terminal part missing in the crystal structure (Fig. 2*B* and *SI Appendix*, Fig. S2). The phosphorylation sites are located in three distinct regions, with five in the kinase core domain (S731, S751, S754, T760, and S807), four sites in the border region between the kinase domain and the distal C-terminal tail, which we named the “alpha-I motif” (S877, S885, S889, and S893), and seven sites in the distal C-terminal tail region (S903, T905, S906, S910, T911, S916, and S918).

### Phosphorylation of the Alpha-I Motif in SYMRK Is Essential for Root Nodule Symbiosis.

To understand which phosphorylation sites outside the kinase core domain are important for root nodule symbiosis, we tested the complementation ability of SYMRK receptor variants expressed from the native promoter and terminator in *Agrobacterium rhizogenes*-induced *symrk* mutant plant roots. As most phosphorylation sites are located in the flexible C-terminus, we first tested whether removal of this region would lead to a symbiotic phenotype (Fig. 2*C*). SYMRK-Δ878-923, lacking the entire C-terminus containing the alpha I-motif, was unable to complement *symrk* mutant plants, as no pink infected nodules were observed when these plants were incubated with the symbiont of *Lotus*, *Mesorhizobium loti* (*M. loti*) (Fig. 2*C*). To test whether phosphorylation is essential for the function of the C-terminus, we created a SYMRK-13A variant where all identified phosphorylation sites plus two putative sites identified by conservation analysis (S877, S885, S889, S893, S903, T905, S906, T907, S910, T911, T914, S916, and S918) were mutated into alanines. The SYMRK-13A variant failed to complement *symrk* (Fig. 2*C*), indicating that phosphorylation events in the C-terminus are essential for the function of SYMRK in root nodule symbiosis. To understand which part of the C-terminus is essential, we created SYMRK-9A, where the nine distal phosphorylation sites were substituted with alanines (S903, T905, S906, T907, S910, T911, T914, S916, and S918). SYMRK-9A showed full complementation of *symrk* (Fig. 2*C*), indicating that the nine distal phosphorylation sites in the tail region are not required for nodulation. Next, we tested the four proximal phosphorylation sites (S877, S885, S889, and S893) and found that SYMRK-4A failed to form nodules (Fig. 2*C*), showing that one or more of the four serines are essential for root nodule symbiosis. The alpha-I motif function is also independent of the distal tail region, since *symrk* mutants transformed with the deletion construct SYMRK-Δ903-923, which lacks the last twenty residues but still contains the alpha-I motif, formed nodules to the same extent as wild-type SYMRK (Fig. 2*C*). To show that the lack of complementation was not the result of reduced protein stability or activity, we recombinantly expressed and purified SYMRK-WT and SYMRK-4A together with a catalytically inactive SYMRK-K622E control (12) (*SI Appendix*, Fig. S1). All purified proteins eluted as single monodisperse protein peaks on gel filtration (Fig. 2*D*) and showed similar thermostability, and all proteins except the catalytically inactive SYMRK-K622E control showed ATP-binding capacity (Fig. 2*E*). Kinase activity assays with γ-^32^P-labeled ATP showed that SYMRK-4A and SYMRK-WT are active kinases capable of both autophosphorylation and transphosphorylation of a Myelin basic protein (MBP) substrate (Fig. 2*F*). To determine whether the phosphoablations in the alpha-I motif or its removal influenced protein localization, we expressed SYMRK versions with a mCherry tag in *Nicotiana benthamiana.* All tested receptor variants showed plasma membrane localization similar to that seen for the wild-type SYMRK-WT control (*SI Appendix*, Fig. S3). Together, our results show that phosphorylation of one or more sites in the alpha-I motif is essential for the role of SYMRK in nodulation.

### Organogenesis Is Driven by Phosphorylation of the Alpha-I Motif.

**Fig. 3.**
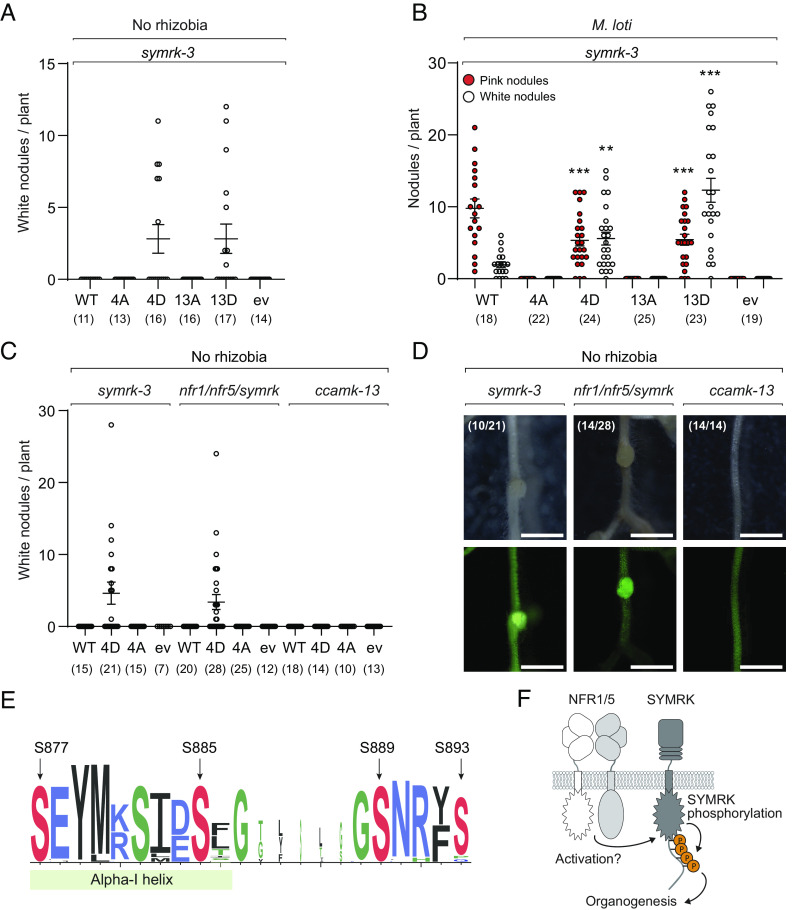
Organogenesis is driven by phosphorylation of the alpha-I motif. (*A*) Number of white (uninfected) nodules on hairy roots expressing the indicated SYMRK constructs expressed under the native promotor in *symrk* mutant plants, 4 wk after emerging of transgenic roots. Numbers below graph specify number of plants for the specific construct (*B*) Number of pink (infected) and white (uninfected) nodules on hairy roots expressing the indicated SYMRK constructs, 4 wk after inoculation with rhizobia. ev is a vector without SYMRK. Statistic: Dunnett’s multiple comparisons test, **P < 0.01, ***P < 0.001. Numbers below graph specify the number of plants for the specific construct. (*C*) Number of white (uninfected) nodules on hairy roots expressing the indicated SYMRK constructs, 4 wk after emerging of transgenic roots. Numbers below graph specify number of plants for the specific construct. (*D*) Representative pictures of roots from (*C*), *Top*: bright field, *Bottom*: Nuclear-localized yellow fluorescent protein (YFP) as transformation marker. (*E*) Sequence logo (Weblogo) showing the conservation of the alpha-I motif in SYMRK proteins. The conserved phosphorylated serines S877, S885, S889, and S893 are indicated. (*F*) Model of signal progression from NFR1/5 via an unknown mechanism leading to phosphorylation of SYMRK and progression of organogenesis via CCaMK.

Since the phosphorylation sites in the alpha-I motif of SYMRK are crucial for nodulation, we investigated whether a gain-of-function SYMRK receptor could activate symbiosis signaling. We replaced the four serines with phosphomimetic aspartates and observed that SYMRK-4D expression in a *symrk* mutant background formed white uninfected nodules in the absence of rhizobia (Fig. 3*A*). Likewise, a phosphomimetic variant for all 13 phosphorylation sites (SYMRK-13D) activates organogenesis in *symrk* mutants without rhizobia, while the phosphoablative construct with alanines (SYMRK-13A) did not (Fig. 3*A*). To understand whether the spontaneously formed nodules were an effect of dysregulated kinase activity, we purified SYMRK-4D and found it to be monodisperse, thermostable, ATP binding, and catalytically active (Fig. 2 *D*–*F* and *SI Appendix*, Fig. S1). Furthermore, SYMRK-4D was correctly localized to the plasma membrane when overexpressed in *N. benthamiana,* similar to SYMRK-WT (*SI Appendix*, Fig. S3). The morphology of the nodules formed on roots complemented with the gain-of-function SYMRK constructs was indistinguishable from the nodules formed on roots expressing wild-type SYMRK-WT (*SI Appendix*, Fig. S4). Since SYMRK is important not only for organogenesis but also for the infection program, we tested whether plants expressing the gain-of-function SYMRK-4D can still be infected by rhizobia. When expressed in transgenic roots, the SYMRK-4D driven by the native *SymRK* promoter produced pink, infected nodules when rhizobia were present (Fig. 3*B*), indicating that a phosphomimetic SYMRK can rescue not only organogenesis but also infection in a *symrk* mutant background. However, the number of infected nodules was reduced in SYMRK-4D/13D compared to the wild type, and the number of white uninfected nodules was increased (Fig. 3*B*). We interpret this to be a consequence of miscoordination between infection and organogenesis. To determine the order of events in the signaling pathway from the Nod factor receptors to SYMRK phosphorylation, we investigated whether SYMRK-4D could drive organogenesis independently of NFR1 and NFR5. Indeed, roots expressing SYMRK-4D from the native *SymRK* promoter showed organogenesis in the absence of rhizobia in the *nfr1/nfr5/symrk* mutant background (Fig. 3 *C* and *D*). However, when SYMRK-4D was expressed in a *ccamk* mutant background, no spontaneous organogenesis was observed (Fig. 3 *C* and *D*). To understand whether the activation of organogenesis by SYMRK-4D in the *nfr1/nfr5/symrk* mutant background could bypass the Nod factor receptors and mediate the formation of infected nodules, we treated the roots with either *M. loti* or IRBG74. No infected nodules formed on the roots with either of the bacteria (*SI Appendix,* Fig. S6). Taken together, this shows that SYMRK phosphorylation is an event downstream of Nod factor perception operating via the common symbiosis pathway and that SYMRK phosphorylation cannot override the need for Nod factor receptors during infection.

## Discussion

In this study, we show that the conserved alpha-I motif in the C-terminal region of SYMRK is essential for root nodule symbiosis (Fig. 3*E*). The regulatory role of the C-terminal region is well-described in other plant receptor kinases. Deletion of the C-terminus of *Arabidopsis* BRI1 results in a hyperactive kinase (18). Likewise, it was shown that *Arabidopsis* BAK1 signaling is regulated by a phosphocode in its C-terminus (19). We identified four phosphorylation sites in the alpha-I motif of SYMRK driving organogenesis and found that all sites are conserved in SYMRK homologs from species that engage in both root nodule symbiosis and arbuscular mycorrhizal symbiosis (*SI Appendix*, Fig. S5*A*). The conservation of these sites in plants that do not form root nodule symbiosis indicates that they could be important for arbuscular mycorrhizal symbiosis signaling as well. This observation is in line with a study showing that rice *SymRK,* a monocot outside the nitrogen-fixing clade, can complement the *symrk* mutants when overexpressed and form functional nodules in *Lotus* (20). The alpha-I motif is not present in other well-characterized plant cell-surface receptors (*SI Appendix*, Fig. S5*B*), indicating that it is unique to SYMRK. We found that several of the identified phosphorylation sites in the distal C-terminal region of SYMRK do not function in organogenesis. However, we cannot exclude that these sites regulate other elements of the symbiotic pathways, such as bacterial infection or establishment of arbuscular mycorrhizal symbiosis.

The mechanism of SYMRK activation is possibly trans-autophosphorylation since the alpha-I motif is located within an extended alpha-I helix that sterically would hinder cis-autophosphorylation. A published study supports autophosphorylation as the mechanism since it was shown that overexpression of SYMRK can drive organogenesis in the absence of rhizobia (21). Considering our study, this suggests that simply increasing the density of SYMRK can trigger alpha-I autophosphorylation and signal activation. Despite these data pointing to SYMRK autophosphorylation as the mechanism of signal activation, we do not exclude that other kinases participate.

Our study shows that phosphorylation of SYMRK activates the downstream signaling pathway, but it is still unknown whether the signal of Nod factor perception is transferred from NFR1 and NFR5 to SYMRK by direct interaction or indirectly via a signal mediator protein. Amino acid substitutions in the alpha-I motif do not affect SYMRK kinase activity. We therefore propose a mechanism where phosphorylation of the four serine residues in the alpha-I motif creates a docking site for downstream components, propagating the signal to CCaMK and ultimately activating organogenesis (Fig. 3*F*). In summary, our study provides a step forward in our understanding of nitrogen fixation signaling by revealing the initial phosphorylation events in SYMRK leading to organogenesis. This understanding may help efforts to transfer the nitrogen-fixing trait to non-legume crops for the development of sustainable agriculture.

## Materials and Methods

### Plasmid Construction and Cloning.

Constructs for expressing phosphomimetic or phosphoablative mutants of *SymRK* were generated using synthesized modules of the 5 kbp promoter of *SymRK*, the 300 bp terminator, wild-type, phosphomimetic-, or phosphorylation-ablation mutants of SYMRK (Thermo Fisher Scientific). The expression vector pIV10 (22) was used for the generation of transgenic roots. All modules are assembled as described in ref. 23. All modules are listed in *SI Appendix,* Table S3. Plasmids for *Escherichia coli* expression of SYMRK kinase domains for crystallization, phosphorylation, and ATP binding were synthesized into the pAH10R7Sumo3C vector (24) by GenScript.

### Expression and Purification of Recombinant SYMRK Kinase from *E. coli*.

SYMRK kinase constructs were transformed into *E. coli* Rosetta 2 cells and grown in LB media until OD600 = 0.6. Protein expression was induced by the addition of 0.4 mM IPTG, and cultures were incubated at 18 °C overnight. Protein was captured from cleared *E. coli* lysate on a Protino Ni-NTA column (Macherey-Nagel) equilibrated in buffer A (25 mM HEPES pH 7.5, 500 mM NaCl, 20 mM imidazole, 5 mM β-mercaptoethanol, 5% glycerol) and eluted in buffer B (buffer A supplemented with 500 mM imidazole). Ni-AC eluates were dialyzed overnight at 4 °C in dialysis buffer (25 mM HEPES pH 7.5, 500 mM NaCl, 1 mM MnCl_2_, 5 mM β-mercaptoethanol, 5% glycerol). During dialysis, the samples were dephosphorylated and cleaved by the addition of his-tagged λ-protein phosphatase and protease 3C in a 1:200 molar ratio. Dialysates were purified in a second Ni-AC step to remove cleaved fusion tags and his-tagged enzymes. Protein purification was finalized in two gel filtration steps using first a Superdex 200 increase 10/300 and then a Superdex 75 increase 10/300 columns (Cytiva) in gel filtration buffer (25 mM HEPES pH 7.5, 200 mM NaCl, 5 mM β-mercaptoethanol).

### Crystallization and Structure Determination.

Crystals of the SYMRK core kinase domain (578 to 877) were obtained using a sitting drop vapor diffusion system at 5 to 10 mg/mL in 1.26 M ammonium sulphate, 0.1 M Tris-HCl pH 8.5. Crystals were cryoprotected by incremental soaking steps in mother liquor containing 10 to 30% (v/v) ethylene glycol before snap-freezing in liquid nitrogen. Diffraction data to 1.95 Å resolution were obtained at the Deutsches Elektronen-Synchrotron P13 beamline in Hamburg, Germany at a wavelength of 0.9762 Å. Data reduction and scaling were performed in XDS (25) and XSCALE, respectively. The phase problem was solved by molecular replacement in phenix.phaser (26, 27) using a homology model built in SWISS-MODEL (28) based on the crystal structure of *Arabidopsis* BIK1 kinase domain (PDB: 5TOS) (14) as a search model. The structure of the three molecules in the asymmetric unit was built in Coot (29) and coordinates and B-factors were refined in phenix.refine (27). Data collection and refinement statistics are reported in *SI Appendix,* Table S1. The ATP molecule in Fig. 1*A* was docked into the binding site by superposition of the Protein kinase A structure (PDB: 1ATP) onto the SYMRK kinase structure.

### Protein Structure Modeling.

The SYMRK intracellular domain including residues 545 to 923 was modeled using the Colabfold implementation of Alphafold2 (30, 31) with three recycles. A MSA containing approximately 20,000 sequences was assembled using mmseqs2 with uniref and environmental databases. No template was used in the modeling process. The resulting five models had high global predicted local distance difference tests (pLDDT) scores of ca. 80, with the core kinase having the highest pLDDT scores and the N-terminal and C-terminal ends being predicted to be disordered. The best model had an overall pLDDT score of 80.9 and was used for further analysis. The composite model of the crystal structure and alpha-I helix/C-terminal tail to visualize phosphorylation sites was made in Coot (29) by superimposition and appending the parts missing from the crystal structure (residues 878 to 923). Structural analysis and figures in the paper were made using chain A of SYMRK in PyMOL version 2.4.1 (Schrödinger LLC).

### Sample Preparation and Phosphopeptide Enrichment for Mass Spectrometry.

Four independent replicas of purified SYMRK were used for phospho-enrichment. Phosphorylation was carried out in buffer (200 mM NaCl, 50 mM TRIS pH 8, 2.5 mM DTT, 20 µM ATP, 5 mM MnCl_2,_ 5 mM MgCl_2_) in a volume of 10 µL with 10 µg SYMRK protein. Reactions were incubated at room temperature for 1 h before flash-freezing in liquid nitrogen. Digest was performed in 250 µL trypsin buffer (50 mM ammonium bicarbonate, 1% SDC pH 8.5) incubated for 10 min at 80 °C followed by 10 min on ice, and 3% M/M Trypsin/Lys-C Mix (Promega) was added. Digestions were performed at 37 °C under shaking for 18 h in the dark. The digestion was terminated by the addition of TFA to 1%. The reaction was cleared by centrifugation for 10 min at 20,000 rpm and the supernatant was used for desalting. For desalting, 200 µL tips with C18 membrane and OLIGO R3 reversed phase resin (Thermo scientific) was employed for desalting and samples were washed twice with 100 µL 1% formic acid and eluted in 50 µL 50% acetonitrile + 0.1% formic acid. Eluted peptides were dried using a speedvac and resuspended in 50 µL 0.1% formic acid. Phospho-enrichment was performed using MagReSYN Zr IMAC beads (Resynbio) (10 times binding capacity to protein amount) with a Kingfisher robot (Thermo scientific) (17). Resuspended peptides + MagReSYN Zr IMAC bead were incubated in 80% acetonitrile, 5% TFA, 0,1M GA for 20 min at room temperature with vigorous shaking, then sequentially washed for 1 min with 80% acetonitrile, 5% TFA, 0,1M GA followed by wash with 80% acetonitrile, 1% TFA and finally with 10% acetonitrile, 0.2% TFA. Purified phosphopeptides were eluted from the beads by 10 min incubation in 1.25 M NH_2_OH. Samples were dried using a speedvac and resuspended in 12 µL 0.1% formic acid prior liquid chromatography with tandem mass spectrometry (LC-MS-MS) analysis (17).

### LC-MS/MS Analysis.

Phosphopeptides were analyzed with the Dionex Ultimate 3000 RSLCnano system coupled online to an Orbitrap Fusion Lumos® tribrid mass spectrometer (Thermo Scientific) (17). Injected peptides were online desalted using a C18 PepMapTM 100 trap column (300 µm i.d. × 5 mm, 5 µm, 100 Å, Thermo Scientific) for 3 min using 2% MeCN + 0.1% FA. Trapped peptides were gradient eluted and separated on a 75 µm i.d. × 18 cm column packed inhouse with ReproSil-Pur 120 C18 1.9 µm particles (Maisch) at a flowrate of 250 nL/min with a 24 min linear gradient of 2 to 34% solvent B (95% MeCN + 0.1% FA) against solvent A (100% H20 + 0.1% FA). The mass spectrometer was set to Data-Dependent Acquisition on positive ion mode. Precursor ion (MS) was analyzed in the Orbitrap and acquired in the range m/z 350 to 1,600 (2+ to 4+ ion charge state) at a mass resolution setting of 120,000 at m/z 400 in profile mode with an AGC target of 1 × 10^6^. The most intense precursor ions were automatically selected for fragmentation with higher-energy collisional dissociation at 34% collision energy and dynamically excluded for 15 s during a cycle time of 2 s. Fragments were analyzed in the Orbitrap at a mass resolution setting of 15,000 at m/z 400 in centroid mode.

### NanoDSF ATP Binding Assay.

*E. coli* expressed SYMRK proteins in gel filtration buffer were supplemented with 5 mM MgCl_2_ and 1 mM ATP for 15 min on ice before measurement of three technical replicates of each sample on a Prometheus NT.48 Series nanoDSF Grade Standard Capillaries (NanoTemper Technologies). ATP binding was assayed by proxy of thermal stabilization in a nano differential scanning fluorimetry (NanoDSF) experiment using a Prometheus Panta (NanoTemper Technologies). SYMRK samples were incubated over a temperature gradient from 25 °C to 95 °C, with a 1 °C/min increment, and protein melting was measured by intrinsic fluorescence emissions at 330/350 nm.

### Kinase Activity Assay.

Radioactive kinase assays were performed in 10 µL reaction mixtures using 3 µg SYMRK and 3 µg MBP in gel filtration buffer. Phosphorylation was started by the addition of 5 mM MgCl_2_, 100 nCi γ-^32^P-labeled ATP, and samples were incubated 1 h at room temperature. Reactions were stopped by the addition of SDS loading dye and 95 °C incubation before samples were assayed by SDS-PAGE. The SDS-PAGE gel was incubated on an Autoradiography Hypercassette (Amersham/Cytiva) overnight and the radiograph was developed using a Typhoon FLA 9500 (Amersham/Cytiva).

### Plant Materials and Growth Conditions.

All hairy root experiments were carried out in *symrk-3*, *nfr1/nfr5/symrk*, or *ccamk-13* mutant backgrounds. Seeds were scarified using sandpaper followed by surface sterilization by 15 min in 1% sodium hypochlorite, afterward washed 5 times in water, and incubated rotating at 4 °C overnight. The following day the seeds were set for germination on wet filter paper for 4 d. Four-day-old seedlings were transferred to square agar plates with Gamborg’s B5 nutrient solution (Duchefa Biochemie) and 0.8% Gelrite (Duchefa Biochemie). *A. rhizogenes* AR1193 carrying the indicated constructs were used for root transformation of 6-d-old seedlings by punching the hypocotyl with a syringe needle and placing a drop of bacteria on top of the wound. Seedlings and bacteria were incubated at 21 °C 2 d in the dark before growing under 16/8-h light/dark conditions for 3 wk. Non-transformed roots were removed, and plants were transferred to pots with lightweight expanded clay aggregate (LECA, 2 to 4 mm; Saint-Gobain Weber A/S) supplemented with B&D nutrient solution (32) as described in ref. 33. One week after transferring to pots, plants were inoculated with *M. loti* MAFF303099 expressing DsRED bacteria (34) OD_600_ = 0.01 and grown for 4 wk before harvest.

### Transient Expression in *N. benthamiana*.

Transformation was performed as described previously (35). In brief: *Agrobacterium tumefaciens* bacteria were resuspended in infiltration solution (10 mM MgCl_2_, 10 mM MES, 150 µM acetosyringone pH 5.6) to an OD600 = 0.1, followed by incubation in the dark for 2 h. The bacteria solution was infiltrated into the leaves with a blunt-end syringe and grown for 3 d.

### Microscopy.

Transgenic roots expressing indicated SYMRK versions or an empty vector (ev) were imaged using a Leica FluoStereo M165FC microscope equipped with a Leica DFC310 FX camera. Expression of wild-type, phosphoablative, and phosphomimetic versions fused to mCherry in *N. benthamiana* were imaged using 561 nm excitation with 571 to 642 nm emission on a Zeiss LSM 780 confocal microscope.

### Sequence Alignments.

Sequences and IDs were obtained through UniProt. Full-sequence alignments were performed in CLC Main Workbench 22 (QIAGEN). Sequence logo of the alpha-I motif was created using Weblogo3. The following IDs were used for alignment of SYMRK sequences: *L. japonicus*—Q8LKX1, *Medicago truncatula*—Q8L4H4, *Glycine max*—K7K1C2, *Cicer arietinum*—A0A1S2YZW4, *Arachis hypogaea*—A0A444WW15, *Pisum sativum*—Q8LKZ1, *Cajanus cajan*—A0A151TH72, *Phaseolus vulgaris*—V7CLT8, *Vigna radiata*—A0A1S3VS57, *Vigna angularis*—A0A0S3T2F3, *Parasponia andersonii*—A0A2P5AQR8, *Hordeum vulgare*—A0A8I6XF58, *Oryza sativa*—Q7F1I0, *Zea mays*—Q208N5, *Ricinus communis*—B9SMK2, *Manihot esculenta*—A0A2C9UVV4, *Citrus sinensis*—A0A067GG96, *Populus trichocarpa*—A0A2K1ZLW1, *Juglans regia*—A0A6P9FAD7, *Solanum lycopersicum*—Q2TDW9, *Prunus dulcis*—A0A5E4FGK8, *Prunus persica*—A0A251NTL9, *Rosa chinensis*—A0A2P6RPN3, and *Cucumis sativus*—A0A0A0KDC4. The following IDs were used for alignment of *Lj*SYMRK with other RLKs of *L. japonicus* or *A. thaliana*: *Lj*NFR5—Q70KR1, *Lj*NFR1—Q70KR8, *Lj*CERK6—D3KTZ6, *At*CERK1—A8R7E6, *At*BRI1—O22476, *At*BAK1—Q94F62, and *At*FLS2—Q9FL28.

## Supplementary Material

Appendix 01 (PDF)

## Data Availability

Atomic coordinates and structure factors for the SYMRK kinase are deposited in the Protein Data Bank (8PEH) (36).

## References

[1] G. E. D. Oldroyd, Speak, friend, and enter: Signalling systems that promote beneficial symbiotic associations in plants. Nat. Rev. Microbiol. **11**, 252–263 (2013). 23493145 10.1038/nrmicro2990

[2] E. B. Madsen , A receptor kinase gene of the LysM type is involved in legume perception of rhizobial signals. Nature **425**, 637–640 (2003). 14534591 10.1038/nature02045

[3] K. Gysel , Kinetic proofreading of lipochitooligosaccharides determines signal activation of symbiotic plant receptors. Proc. Natl. Acad. Sci. U.S.A. **118**, e2111031118 (2021). 34716271 10.1073/pnas.2111031118 PMC8612216

[4] Z. Bozsoki , Ligand-recognizing motifs in plant LysM receptors are major determinants of specificity. Science **369**, 663–670 (2020). 32764065 10.1126/science.abb3377

[5] S. Radutoiu , Plant recognition of symbiotic bacteria requires two LysM receptor-like kinases. Nature **425**, 585–592 (2003). 14534578 10.1038/nature02039

[6] S. Stracke , A plant receptor-like kinase required for both bacterial and fungal symbiosis. Nature **417**, 959–962 (2002). 12087405 10.1038/nature00841

[7] H. Rübsam , Nanobody-driven signaling reveals the core receptor complex in root nodule symbiosis. Science **379**, 272–277 (2023). 36656954 10.1126/science.ade9204

[8] R. Huisman, R. Geurts, A roadmap toward engineered nitrogen-fixing nodule symbiosis. Plant Commun. **1**, 100019 (2020). 33404552 10.1016/j.xplc.2019.100019 PMC7748023

[9] L. Tirichine , Deregulation of a Ca^2+^/calmodulin-dependent kinase leads to spontaneous nodule development. Nature **441**, 1153–1156 (2006). 16810257 10.1038/nature04862

[10] J. Lévy , A putative Ca^2+^ and calmodulin-dependent protein kinase required for bacterial and fungal symbioses. Science **303**, 1361–1364 (2004). 14963335 10.1126/science.1093038

[11] S. Singh, K. Katzer, J. Lambert, M. Cerri, M. Parniske, CYCLOPS, A DNA-binding transcriptional activator, orchestrates symbiotic root nodule development. Cell Host Microbe **15**, 139–152 (2014). 24528861 10.1016/j.chom.2014.01.011

[12] S. Yoshida, M. Parniske, Regulation of plant symbiosis receptor kinase through serine and threonine phosphorylation. J. Biol. Chem. **280**, 9203–9209 (2005). 15572355 10.1074/jbc.M411665200

[13] J. A. Perry , A TILLING reverse genetics tool and a web-accessible collection of mutants of the legume Lotus japonicus. Plant Physiol. **131**, 866–871 (2003). 12644638 10.1104/pp.102.017384 PMC1540285

[14] N. K. Lal , The receptor-like cytoplasmic kinase BIK1 localizes to the nucleus and regulates defense hormone expression during plant innate immunity. Cell Host Microbe **23**, 485–497.e5 (2018). 29649442 10.1016/j.chom.2018.03.010 PMC6266874

[15] Z. Wang , Crystal structures of IRAK-4 kinase in complex with inhibitors: A serine/threonine kinase with tyrosine as a gatekeeper. Structure **14**, 1835–1844 (2006). 17161373 10.1016/j.str.2006.11.001

[16] L. Yan , Structural basis for the impact of phosphorylation on the activation of plant receptor-like kinase BAK1. Cell Res. **22**, 1304–1308 (2012). 22547027 10.1038/cr.2012.74 PMC3411173

[17] I. Arribas Diez , Zirconium(IV)-IMAC revisited: Improved performance and phosphoproteome coverage by magnetic microparticles for phosphopeptide affinity enrichment. J. Proteome Res. **20**, 453–462 (2021). 33226818 10.1021/acs.jproteome.0c00508

[18] X. Wang , Autoregulation and homodimerization are involved in the activation of the plant steroid receptor BRI1. Dev. Cell **8**, 855–865 (2005). 15935775 10.1016/j.devcel.2005.05.001

[19] A. Perraki , Phosphocode-dependent functional dichotomy of a common co-receptor in plant signalling. Nature **561**, 248–252 (2018). 30177827 10.1038/s41586-018-0471-x PMC6250601

[20] H. Li , Domain swap approach reveals the critical roles of different domains of symrk in root nodule symbiosis in Lotus japonicus. Front. Plant Sci. **9**, 697 (2018). 29988452 10.3389/fpls.2018.00697 PMC6024595

[21] M. K. Ried, M. Antolín-Llovera, M. Parniske, Spontaneous symbiotic reprogramming of plant roots triggered by receptor-like kinases. Elife **3**, 1–17 (2014). 10.7554/eLife.03891 PMC4243133 25422918

[22] J. Stougaard, *Agrobacterium rhizogenes* as a vector for transforming higher plants. Methods Mol. Biol. **49**, 49–61 (1995). 8563829 10.1385/0-89603-321-X:49

[23] E. Weber, C. Engler, R. Gruetzner, S. Werner, S. Marillonnet, A modular cloning system for standardized assembly of multigene constructs. PLoS One **6**, e16765 (2011). 21364738 10.1371/journal.pone.0016765 PMC3041749

[24] K. R. Andersen , Scaffold nucleoporins Nup188 and Nup192 share structural and functional properties with nuclear transport receptors. Elife **2**, e00745 (2013). 23795296 10.7554/eLife.00745 PMC3679522

[25] W. Kabsch, Integration, scaling, space-group assignment and post-refinement. Acta Crystallogr. D Biol. Crystallogr. **66**, 133–144 (2010). 20124693 10.1107/S0907444909047374 PMC2815666

[26] A. J. McCoy , Phaser crystallographic software. J. Appl. Crystallogr. **40**, 658–674 (2007). 19461840 10.1107/S0021889807021206 PMC2483472

[27] D. Liebschner , Macromolecular structure determination using X-rays, neutrons and electrons: Recent developments in Phenix. Acta Crystallogr. D Struct. Biol. **75**, 861–877 (2019). 31588918 10.1107/S2059798319011471 PMC6778852

[28] A. Waterhouse , SWISS-MODEL: Homology modelling of protein structures and complexes. Nucleic Acids Res. **46**, W296–W303 (2018). 29788355 10.1093/nar/gky427 PMC6030848

[29] P. Emsley, B. Lohkamp, W. G. Scott, K. Cowtan, Features and development of Coot. Acta Crystallogr. D Biol. Crystallogr. **66**, 486–501 (2010). 20383002 10.1107/S0907444910007493 PMC2852313

[30] J. Jumper , Highly accurate protein structure prediction with AlphaFold. Nature **596**, 583–589 (2021). 34265844 10.1038/s41586-021-03819-2 PMC8371605

[31] M. Mirdita , ColabFold: Making protein folding accessible to all. Nat. Methods **19**, 679–682 (2022). 35637307 10.1038/s41592-022-01488-1 PMC9184281

[32] W. J. Broughton, M. J. Dilworth, Control of leghaemoglobin synthesis in snake beans. Biochem. J. **125**, 1075–1080 (1971). 5144223 10.1042/bj1251075 PMC1178271

[33] S. Ferguson , A simple and efficient protocol for generating transgenic hairy roots using Agrobacterium rhizogenes. PLoS One **18**, e0291680 (2023). 37910566 10.1371/journal.pone.0291680 PMC10619795

[34] T. Maekawa , Gibberellin controls the nodulation signaling pathway in *Lotus japonicus*. Plant J. **58**, 183–194 (2009). 19121107 10.1111/j.1365-313X.2008.03774.x

[35] D. Arora , Establishment of proximity-dependent biotinylation approaches in different plant model systems. Plant Cell **32**, 3388–3407 (2020). 32843435 10.1105/tpc.20.00235 PMC7610282

[36] M. M. M. Noergaard, K. Gysel, S. B. Hansen, K. R. Andersen, Crystal structure of Lotus japonicus SYMRK kinase domain D738N. Protein Data Bank. https://www.rcsb.org/structure/8PEH. Deposited 14 June 2023.

