# Supplementary material for: Phosphorylation of the alpha-I motif in SYMRK drives root nodule organogenesis
Source: Proc Natl Acad Sci U S A. 2024 Feb 16;121(8):e2311522121. doi: 10.1073/pnas.2311522121 (PMC10895371; doi:10.1073/pnas.2311522121)
Supplement: Supplementary file 1 — Appendix 01 (PDF) [file pnas.2311522121.sapp.pdf]

PNAS

Supporting Information for

Phosphorylation of the alpha-I motif in SYMRK drives root nodule organogenesis

Nikolaj B. Abel[a,1,*], Malita M. M. Nørgaard[a,1], Simon B. Hansen[a], Kira Gysel[a], Ignacio A. Diez[b], Ole N. Jensen[b], Jens Stougaard[a] & Kasper R. Andersen[a,*]

*Nikolaj B. Abel and Kasper R. Andersen
Email: nikolaj.abel@mbg.au.dk and kra@mbg.au.dk

This PDF file includes:

Figures S1 to S6
Tables S1 to S3

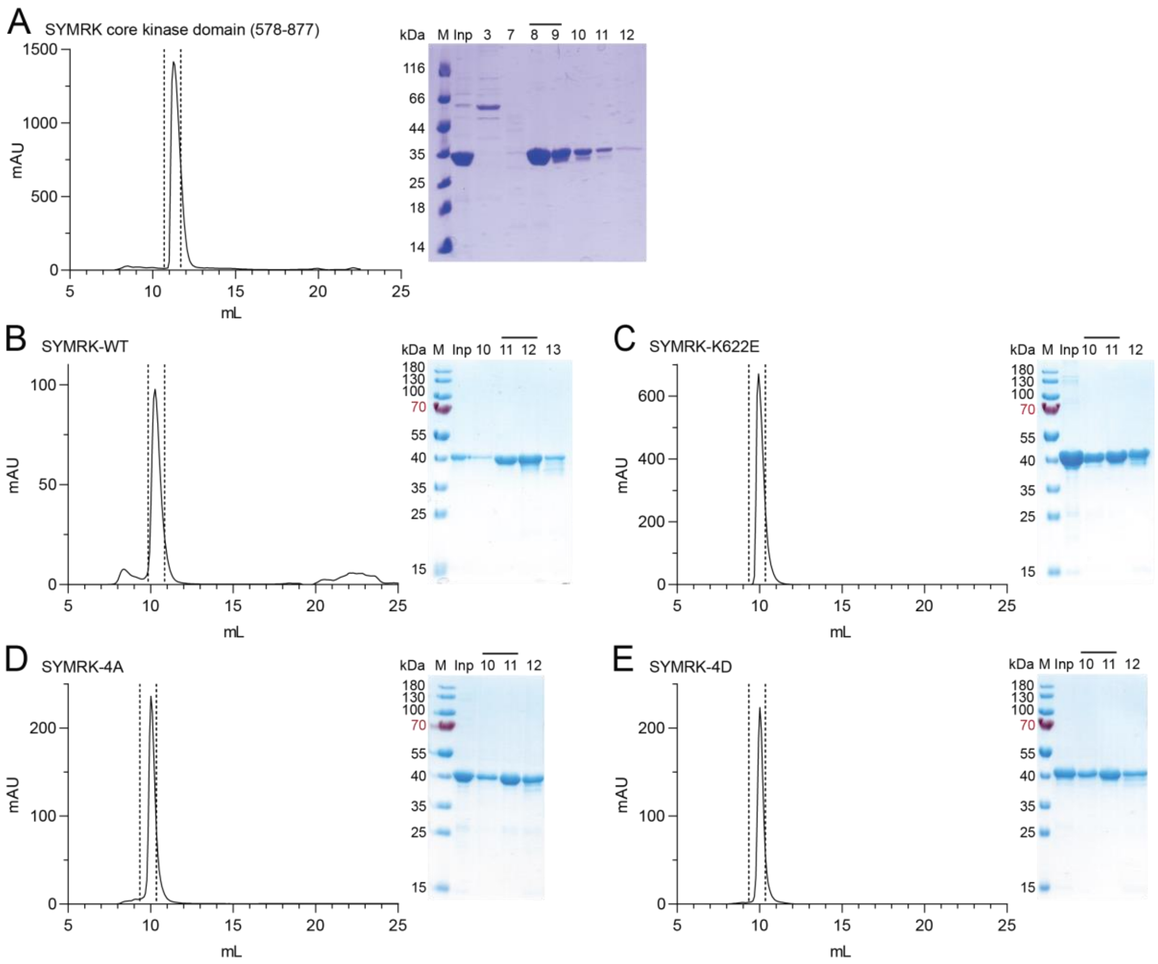

Fig. S1. (*A*-*E*) Purification and gel filtration profiles of SYMRK intracellular domains and associated SDS-PAGE analysis with fractions used for experiments indicated by a black bar. Pooled fractions are indicated by dashed lines in chromatograms and horizontal black lines above fraction lanes in SDS-PAGE gels. All preparations show high final purity with expected molecular weights as determined by SDS-PAGE analysis. M = molecular weight marker, Inp = input sample.

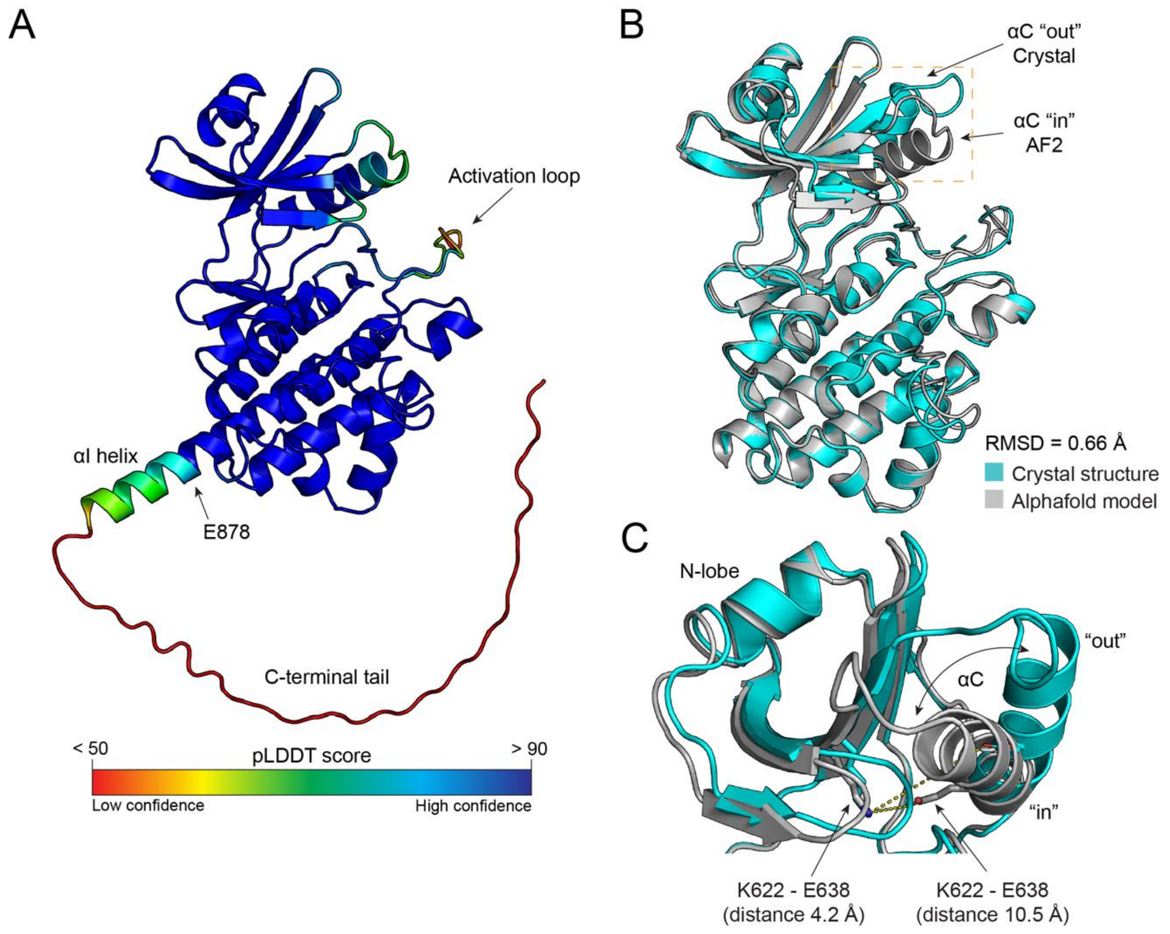

Fig. S2. Alphafold2 models of the SYMRK kinase domain and comparison to the crystal structure. (*A*) Cartoon representation of the Alphafold2 model of SYMRK kinase domain (residues 578-923) including the activation loop and full C-terminus. Residues 878-923 were used to generate the composite model shown in Fig. 2*B*. Colors and scalebar indicate the Alphafold2 confidence pLDDT value (predicted local distance difference test) per residue. (*B*) Superposition of the SYMRK kinase domain crystal structure and the core kinase domain of the AF2 prediction model (residues 578-877) show an almost identical fold with a root mean square deviation (RMSD) of 0.66 Å between the two models. The largest difference is a conformational change in the αC, which is in an inactive "out" conformation in the crystal structure, whereas Alphafold2 predicts the active "in" position. (*C*) Zoomed view of the kinase N-lobe. In the crystal structure, the salt bridge between K622 and E638 is broken, whereas Alphafold2 models an active state with the K622-E638 salt bridge formed.

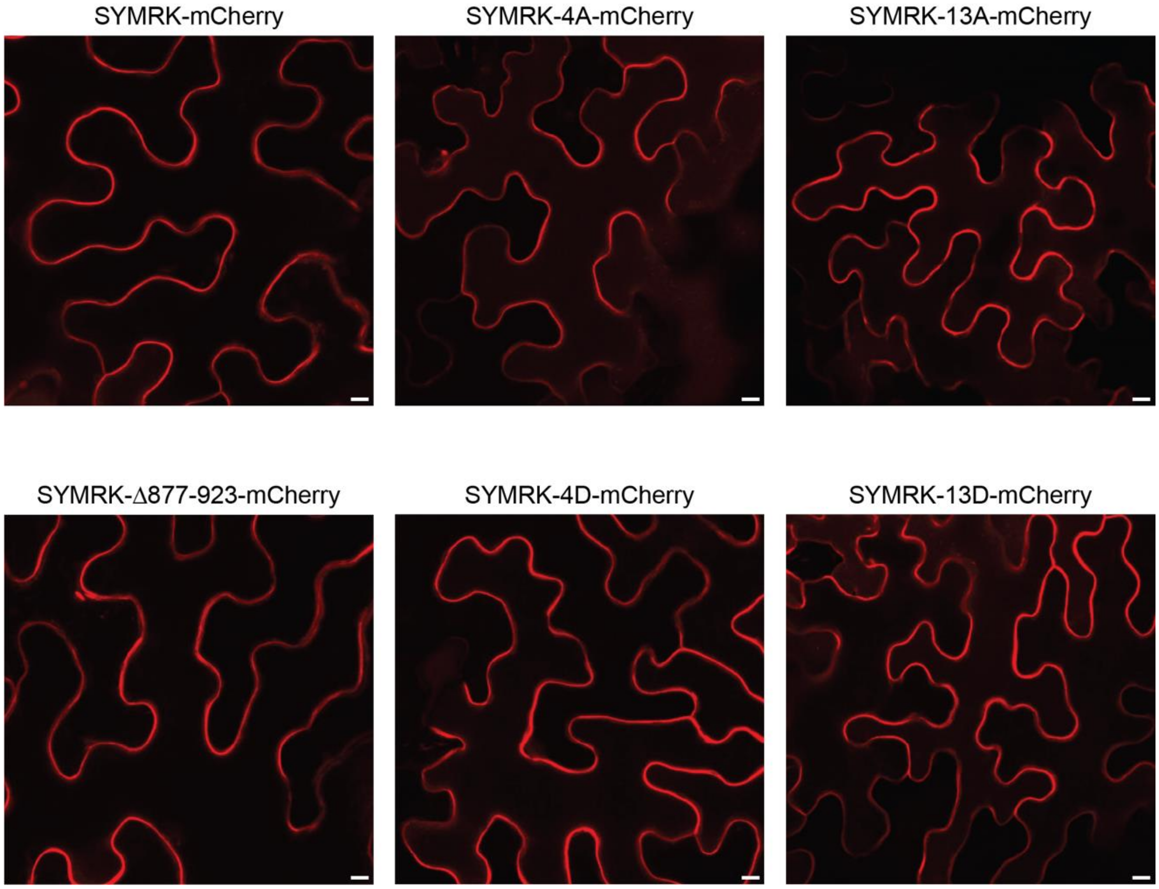

Fig. S3. Plasma membrane localization of indicated SYMRK constructs with mCherry tag expressed in *Nicotiana benthamiana* leaves. Scale bars indicate 10 μm.

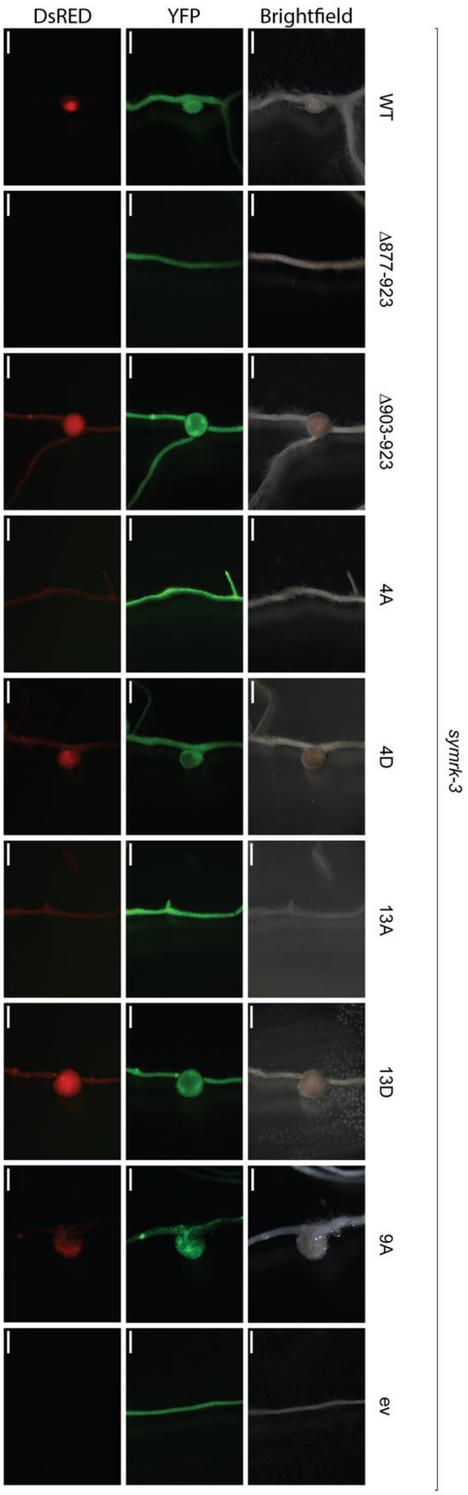

Supplementary figure 4 Representative pictures of roots and nodules on hairy roots expressing the indicated SYMRK constructs under the native promoter in *symrk-3* mutant plants, four weeks after inoculation with rhizobia. Nuclear-localized YFP was used as a transformation marker; rhizobia were labeled with DsRED. Empty vector (ev) is a control vector without SYMRK. Scale bars indicate 1 mm.

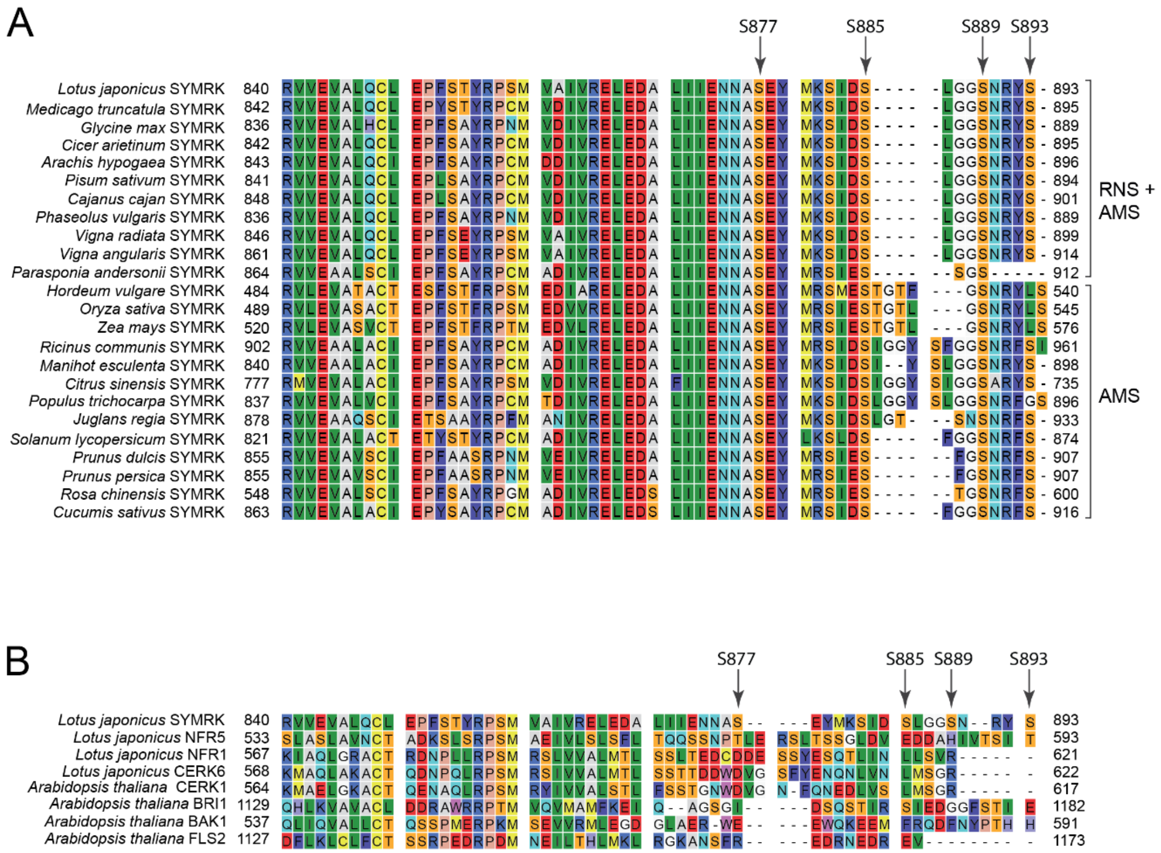

Fig. S5. (*A*) Alignment of SYMRK sequences from species able to engage in both root nodule symbiosis (RNS) and arbuscular mycorrhizal symbiosis (AMS), or only in AMS. Arrows denote positions of *Lotus japonicus* SYMRK S877, S885, S889 and S893. Numbers to the left and right of the alignment indicate respective amino acid positions. (*B*) Alignment of *Lotus japonicus* SYMRK with other well-characterized plant cell-surface receptors: *Lj*NFR5, *Lj*NFR1, *Lj*CERK6, *At*CERK1, *At*BRI1, *At*BAK1, *At*FLS2. Arrows denote positions of *Lotus japonicus* SYMRK S877, S885, S889 and S893. Numbers to the left and right of the alignment indicate respective amino acid positions.

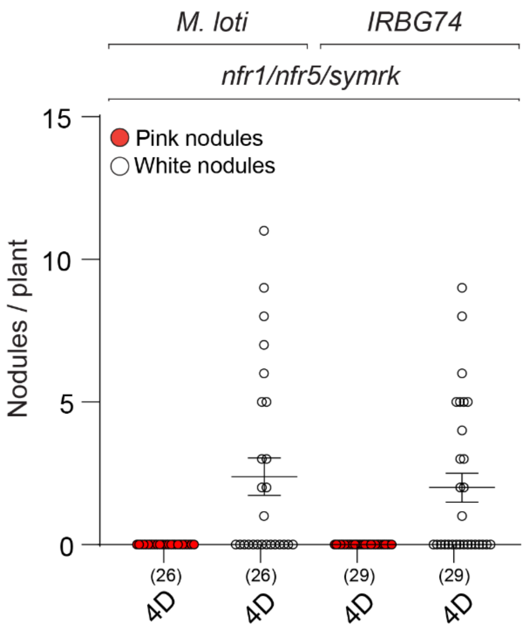

Fig. S6. Number of pink (infected) and white (uninfected) nodules on hairy roots expressing the SYMRK-4D constructs in *nfr1/nfr5/symrk* mutant background, four weeks after inoculation with *M. loti* or IRBG74. Numbers below graph specify the number of plants for the specific construct.

Table S1.
Data collection and refinement statistics

	LjSYMRK (587-877) D738N
**Data collection statistics**	
X-ray source	P13 (DESY, PETRA III)
Temperature (K)	100
Wavelength (Å)	0.9762
Resolution range (Å)	41.59 - 1.95 (2.02 - 1.95)
Space group	C 1 2 1
a, b, c (Å)	144.87, 77.96, 101.40
α, ß, γ (°)	90, 90.15, 90
Unique reflections	80272 (7828)
Completeness (%)	97.40 (95.95)
Multiplicity	6.9 (6.9)
Mean I/σ(I)	9.16 (1.31)
Wilson B-factor (Å²)	34.96
R_{meas}	0.133 (1.695)
R_{pim}	0.050 (0.6385)
CC1/2	0.997 (0.638)
**Refinement statistics**	
Reflections (R_{work})	80243 (7814)
Reflections (R_{free})	3738 (348)
R_{work}	0.1955 (0.3582)
R_{free}	0.2306 (0.3745)
**ASU contents and geometry**	
n in asymmetric unit	3
Number of non-hydrogen atoms	7477
macromolecules	6855
solutes	111
solvent	511
Protein residues	859
RMS deviations	
Bond lengths (Å)	0.006
Bond angles (°)	0.78
Ramachandran favored (%)	97.37
Ramachandran allowed (%)	2.63
Ramachandran outliers (%)	0
Rotamer outliers (%)	0.54
Average B-factor (Å²)	48.97
macromolecules	48.55
solutes	70.53
solvent	50.03
Clashscore	4.55

Data were collected from one crystal.
Values in parentheses represent the highest resolution shell.

Table S2.

Confidence	Annotated Sequence	Modifications	Positions in Proteins	Modifications in Proteins	# Missed Cleavages	Theo. MH+ [Da]	Abundances (Grouped): SYMRK	Abundances (Grouped) CV [%]: SYMRK	Found in Sample: [S1] F1: Sample, SYMRK, n/a, n/a	Found in Sample: [S2] F2: Sample, SYMRK, n/a, n/a	Found in Sample: [S3] F3: Sample, SYMRK, n/a, n/a	Found in Sample: [S4] F4: Sample, SYMRK, n/a, n/a	Confidence (by Search Engine): Mascot	Ions Score (by Search Engine): Mascot	Top Apex RT [min]
High	[R].VLPSTTSTAESTITTQSLSHPQPR.[-]	1xPhospho [S17(100)]	Q8LKX1 [900-923]	Q8LKX1 1xPhospho [S916(100)]	0	2619,26617	780828556,4	74,03	High	High	High	High	High	147	13,11
High	[R].VLPSTTSTAESTITTQSLSHPQPR.[-]	2xPhospho [S11(99.3); S17(100)]	Q8LKX1 [900-923]	Q8LKX1 2xPhospho [S910(99.3); S916(100)]	0	2699,2325	8834143261	25,38	High	High	High	High	High	145	13,19
High	[K].VADFGFSKYAPQEGDSYVSLEVR.[G]	1xPhospho [S16(99.6)]	Q8LKX1 [736-758]	Q8LKX1 1xPhospho [S751(99.6)]	1	2644,19669	805727,2058	145,32	High	High	High	High	High	132	15,48
High	[R].VLPSTTSTAESTITTQSLSHPQPR.[-]	3xPhospho [S11(100); S17(100); H20(100)]	Q8LKX1 [900-923]	Q8LKX1 3xPhospho [S910(100); S916(100); H919(100)]	0	2779,19883	6578835846	27,38	High	High	High	High	High	125	13,39
High	[K].RVLPSTTSTAESTITTQSLSHPQPR.[-]	4xPhospho [S5(98.8); T7(97.5); S12(100); S18(100)]	Q8LKX1 [899-923]	Q8LKX1 4xPhospho [S903(98.8); T905(97.5); S910(100); S916(100)]	1	3015,26627	21802434,44	98,36	High	High	Peak Found	High	High	123	13,6
High	[R].VLPSTTSTAESTITTQSLSHPQPR.[-]	4xPhospho [S11(98.8); S17(98.8); S19(100); H20(100)]	Q8LKX1 [900-923]	Q8LKX1 4xPhospho [S910(98.8); S916(98.8); S918(100); H919(100)]	0	2859,16516	898299406,5	71,91	High	High	High	High	High	120	13,49
High	[K].RVLPSTTSTAESTITTQSLSHPQPR.[-]	3xPhospho [S5(98.1); S12(100); S18(100)]	Q8LKX1 [899-923]	Q8LKX1 3xPhospho [S903(98.1); S910(100); S916(100)]	1	2935,29994	174015014	73,03	High	High	High	High	High	120	13,3
High	[K].RVLPSTTSTAESTITTQSLSHPQPR.[-]	2xPhospho [S5(98.7); S18(100)]	Q8LKX1 [899-923]	Q8LKX1 2xPhospho [S903(98.7); S916(100)]	1	2855,33361	151245200	83,36	High	High	High	High	High	119	12,8
High	[K].RVLPSTTSTAESTITTQSLSHPQPR.[-]	1xPhospho [S18(100)]	Q8LKX1 [899-923]	Q8LKX1 1xPhospho [S916(100)]	1	2775,36728	4627818,503	126,32	High	High	High	High	High	115	12,67
High	[K].VADFGFSKYAPQEGDSYVSLEVR.[G]	2xPhospho [S16(100); S19(100)]	Q8LKX1 [736-758]	Q8LKX1 2xPhospho [S751(100); S754(100)]	1	2724,16302	5812461,868	127,39	High	High	High	High	High	113	15,7
High	[K].SSNILLDHSMCAKVADFGFSK.[Y]	1xCarbamidomethyl [C11]; 1xPhospho [S9(100)]	Q8LKX1 [723-743]	Q8LKX1 1xPhospho [S731(100)]	1	2407,08219	309857,9375	135,27	High	High	Peak Found	Not Found	High	92	15,43
High	[K].YAPQEGDSYVSLEVR.[G]	1xPhospho [S8(100)]	Q8LKX1 [744-758]	Q8LKX1 1xPhospho [S751(100)]	0	1792,77894	4466828,228	50,7	High	High	High	High	High	89	14,43
High	[K].SSNILLDHSMCAK.[V]	1xCarbamidomethyl [C11]; 1xPhospho [S9(100)]	Q8LKX1 [723-735]	Q8LKX1 1xPhospho [S731(100)]	0	1555,66444	26511422,28	25,96	High	High	High	High	High	88	13,48
High	[K].YAPQEGDSYVSLEVR.[G]	2xPhospho [S8(100); S11(100)]	Q8LKX1 [744-758]	Q8LKX1 2xPhospho [S751(100); S754(100)]	0	1872,74527	28947236,98	47,99	High	High	High	High	High	85	14,49
High	[K].SIDSLGGSNRYSIVIEK.[R]	1xPhospho [S8(100)]	Q8LKX1 [882-898]	Q8LKX1 1xPhospho [S889(100)]	1	1917,93175	5278612,478	145,29	High	High	High	High	High	84	14,43
High	[K].SSNILLDHSMCAK.[V]	1xCarbamidomethyl [C11]; 1xPhospho [S9(100)]; 1xOxidation [M10]	Q8LKX1 [723-735]	Q8LKX1 1xPhospho [S731(100)]	0	1571,65936	17025106,38	38,39	High	High	High	High	High	77	12,62
High	[K].SIDSLGGSNRYSIVIEK.[R]	2xPhospho [S4(100); S8(100)]	Q8LKX1 [882-898]	Q8LKX1 2xPhospho [S889(100); S893(100)]	1	1997,89808	28922134,58	140,72	High	High	High	High	High	76	14,71
High	[R].DIKSSNILLDHSMCAK.[V]	1xCarbamidomethyl [C14]; 1xPhospho [S12(100)]	Q8LKX1 [720-735]	Q8LKX1 1xPhospho [S731(100)]	1	1911,87041	6668740,36	45,54	High	High	High	High	High	76	13,68
High	[R].VLPSTTSTAESTITTQSLSHPQPR.[-]	5xPhospho [S/T]	Q8LKX1 [900-923]	Q8LKX1 5xPhospho [T/S]	0	2939,13149	3360569,816	165,64	Peak Found	High	Peak Found	High	High	75	12,98
High	[K].SIDSLGGSNRYSIVIEKR.[V]	1xPhospho [S8(100)]	Q8LKX1 [882-899]	Q8LKX1 1xPhospho [S889(100)]	2	2074,03286	19553840,47	168,26	High	High	High	High	High	74	13,7
High	[K].SSNILLDHSMCAKVADFGFSK.[Y]	1xCarbamidomethyl [C11]; 1xPhospho [S9(100)]; 1xOxidation [M10]	Q8LKX1 [723-743]	Q8LKX1 1xPhospho [S731(100)]	1	2423,07711	748751,0153	80,89	High	High	Not Found	Not Found	High	72	14,67
High	[K].SIDSLGGSNRYSIVIEK.[R]	3xPhospho [S4(100); S8(100); S12(100)]	Q8LKX1 [882-898]	Q8LKX1 3xPhospho [S885(100); S889(100); S893(100)]	1	2077,86441	4428219,633	64,1	High	High	High	High	High	70	15,12
High	[R].ELEDALIIENNASEYMK.[S]	1xPhospho [S13(100)]	Q8LKX1 [865-881]	Q8LKX1 1xPhospho [S877(100)]	0	2061,90863	317202,1865	135,31	High	Peak Found	High	High	High	68	16,1
High	[R].DIKSSNILLDHSMCAK.[V]	1xCarbamidomethyl [C14]; 1xPhospho [S12(100)]; 1xOxidation [M13]	Q8LKX1 [720-735]	Q8LKX1 1xPhospho [S731(100)]	1	1927,86533	7038126,046	32,01	High	High	High	High	High	66	12,99
High	[R].DIKSSNILLDHSMCAK.[V]	1xCarbamidomethyl [C14]; 2xPhospho [S5(100); S12(100)]	Q8LKX1 [720-735]	Q8LKX1 2xPhospho [S724(100); S731(100)]	1	1991,83674	2612209,619	53,76	High	High	High	High	High	57	14,05
High	[R].ELEDALIIENNASEYMK.[S]	1xPhospho [S13(100)]; 1xOxidation [M16]	Q8LKX1 [865-881]	Q8LKX1 1xPhospho [S877(100)]	0	2077,90354	788847,3442	42,33	High	High	High	High	High	53	15,35
High	[K].SIDSLGGSNRYSIVIEKR.[V]	2xPhospho [S8(100); S12(100)]	Q8LKX1 [882-899]	Q8LKX1 2xPhospho [S889(100); S893(100)]	2	2153,99919	409685165,4	144,48	High	High	High	High	High	51	14,07
High	[K].SIDSLGGSNR.[Y]	2xPhospho [S4(100); S8(100)]	Q8LKX1 [882-891]	Q8LKX1 2xPhospho [S885(100); S889(100)]	0	1165,42862	621938,4265	31,16	High	High	High	High	High	50	12,72
High	[R].YSIVIEKR.[V]	1xPhospho [S2(100)]	Q8LKX1 [892-899]	Q8LKX1 1xPhospho [S893(100)]	1	1087,55474	1954304,636	63,3	High	High	High	High	High	46	13,08
High	[K].SIDSLGGSNRYSIVIEKR.[V]	3xPhospho [S4(100); S8(100); S12(100)]	Q8LKX1 [882-899]	Q8LKX1 3xPhospho [S885(100); S889(100); S893(100)]	2	2233,96552	115963512	91,43	High	High	High	High	High	46	14,74
High	[R].GTAGYLDPEYYK.[T]	1xPhospho [T2(100)]	Q8LKX1 [759-770]	Q8LKX1 1xPhospho [T760(100)]	0	1456,6032	274203,75	11,15	Not Found	Peak Found	Peak Found	High	High	40	14,71
High	[R].ELEDALIIENNASEYMKSIDSLGGSNRYSIVIEK.[R]	2xPhospho [S18(100); S25(98.7)]; 1xOxidation [M16]	Q8LKX1 [865-898]	Q8LKX1 2xPhospho [S882(100); S889(98.7)]	2	3976,81745	156504,6056	60,08	High	Peak Found	Not Found	Not Found	High	39	15,97
High	[R].TEWSLVEWATPYIR.[G]	1xPhospho [S4(99.5)]	Q8LKX1 [804-817]	Q8LKX1 1xPhospho [S807(99.5)]	0	1830,84623	30532,48438		Not Found	Not Found	High	High	High	35	17,12
High	[K].VRSATSTQGTR.[E]	1xPhospho [S/T]	Q8LKX1 [623-633]		1	1243,57905	301695,8325	71,65	Peak Found	High	Peak Found	Peak Found	High	21	9,01

Phosphopeptides identified on purified SYMRK intracellular domain. Phosphorylation sites identified in four replicas of phospho-enriched SYMRK. Peptides were identified in a minimum of two replicas and sequenced in minimum of one (High confidence, MS/MS).

Table S3.

**Construct name**	**Detailed name**	**Reference**
**For generation of transgenic roots in *Lotus***		
SYMRK-WT	*pSymRK:SymRK(WT)-mCherry-tSymRK / pUBI:tYFP-NLS-t35s*	This study
SYMRK-4A	*pSymRK:SymRK(4A)-mCherry-tSymRK / pUBI:tYFP-NLS-t35s*	This study
SYMRK-4D	*pSymRK:SymRK(4D)-mCherry-tSymRK / pUBI:tYFP-NLS-t35s*	This study
SYMRK-9A	*pSymRK:SymRK(9A)-mCherry-tSymRK / pUBI:tYFP-NLS-t35s*	This study
SYMRK-13A	*pSymRK:SymRK(13A)-mCherry-tSymRK / pUBI:tYFP-NLS-t35s*	This study
SYMRK-13D	*pSymRK:SymRK(13D)-mCherry-tSymRK / pUBI:tYFP-NLS-t35s*	This study
SYMRK-Δ877-923	*pSymRK:SymRK(Δ877-923)-mCherry-tSymRK / pUBI:tYFP-NLS-t35s*	This study
SYMRK-Δ903-923	*pSymRK:SymRK(Δ903-923)-mCherry-tSymRK / pUBI:tYFP-NLS-t35s*	This study
Empty vector	*pUBI:tYFP-NLS-t35s*	This study
**For *N. benthamiana* transformation**		
SYMRK-WT	*p35S:SymRK(WT)-mCherry-t35S*	This study
SYMRK-4A	*p35S:SymRK(4A)-mCherry-t35S*	This study
SYMRK-4D	*p35S:SymRK(4D)-mCherry-t35S*	This study
SYMRK-13A	*p35S:SymRK(13A)-mCherry-t35S*	This study
SYMRK-13D	*p35S:SymRK(13D)-mCherry-t35S*	This study
SYMRK-Δ877-923	*p35S:SymRK(Δ877-923)-mCherry-t35S*	This study
**For protein purification from E.coli**		
SYMRK-WT	*pAH10R7Sumo3C_SymRK (res 545-923)*	This study
SYMRK-K622E	*pAH10R7Sumo3C_SymRK (res 545-923)_K622E*	This study
SYMRK-4A	*pAH10R7Sumo3C_SymRK (res 545-923)_S877A, S885A, S889A, S893A*	This study
SYMRK-4D	*pAH10R7Sumo3C_SymRK (res 545-923)_S877D, S885D, S889D, S893D*	This study
SYMRK crystal D738N	*pAH10R7Sumo3C_SymRK (res 578-877)_D738N*	This study

Constructs used in this study. Native promoter of *SymRK* from *Lotus* (p*SymRK*) SYMRK gene from *Lotus* (*SymRK*), native terminator of *Lotus* (t*SymRK*), ubiquitin promoter from *Lotus* (pUBI), Triple YFP (tYFP), nuclear localization signal (NLS), Promoter of the cauliflower mosaic virus (p35s) and terminator of the cauliflower mosaic virus (t35s).
